# Rectal Atresia—Operative Management with Endoscopy and Transanal Approach: A Case Report

**DOI:** 10.1155/2011/792402

**Published:** 2011-04-21

**Authors:** Pernilla Stenström, Christina Clementson Kockum, Einar Arnbjörnsson

**Affiliations:** Department of Paediatric Surgery, Skåne University Hospital and Lund University, 22185 Lund, Sweden

## Abstract

The aim of this study is to present the technique and outcome of the management of a newborn child with rectal atresia. A girl born with rectal atresia was diagnosed during physical examination and confirmed with X-ray. The anatomic appearance of the external anus, and lower pelvis was normal. The rectal ending was located 2 cm cranial from the anus and the distance between the rectal endings was 2 cm. A colostomy was established. At the age of five months the child was operated on with a rectal anastomosis using the endoscopic and transanal approach. Closure of the colostomy was performed at the age of ten months. The rectal anastomosis was treated with rectal dilatation weekly in order to avoid stricture. The patient was faecally continent at followup one and three months postoperatively. In conclusion, the endoscopic and transanal approach is an alternative to other surgical techniques in the management of rectal atresia.

## 1. Introduction

Rectal atresia is a rare condition, with a reported incidence of 0.3–1.2% of all anorectal malformations [1]. Rectal atresia is combined with well-developed pelvic structures—the anal canal, external sphincter, and internal sphincter are complete. The anal canal and part of the rectum are developed, but they are separated by an atretic segment of rectum [2]. Usually, there is no fistula between the rectum and the urethra or vagina [1, 3]. Different approaches and techniques in the management of this rare problem have been reported (Table 1). In the surgical management of this condition, an ideal operation should preserve the anatomy, leaving a postoperative good result without damage or scars to the pelvic region. Our hypothesis was that an endoscopic and transanal approach could fulfill the mentioned requirements and therefore provide good faecal continence. The aim of this paper is to present the technique and outcome of an endoscopic and transanal approach in the management of rectal atresia.

## 2. Case Report

**Figure 1:**
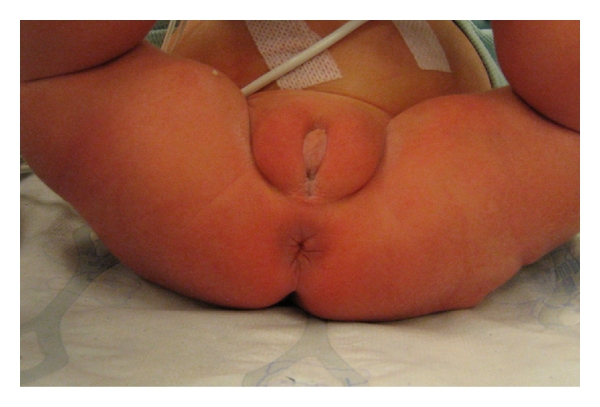
Normal external anatomy at 24 hours.

A 22-hour-old, full-term, 3.7-kg girl, born after an uneventful pregnancy, was admitted for abdominal distension and failure to pass meconium. The genitals and external anus were normal and well formed (Figure 1). 

**Figure 2:**
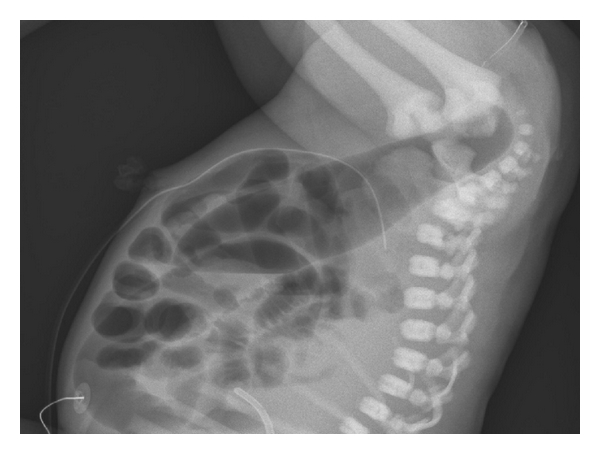
X-ray of abdomen at 24 hours of age before colostomy. A catheter is placed at the bottom of the anal channel.

A digital rectal examination showed a blind ending of the anal canal 2 cm above the dentate line. There was no meconium in the urine. An X-ray of the abdomen showed a blind ending dilated intestine, 2 cm from rectum (Figure 2). The neonate was diagnosed with rectal atresia, of septal type [2]. A sigmoid divided colostomy was performed. Postoperatively the distal segment of the colostomy was cleaned by using an enema from the distal stoma opening once every week in order to avoid fecal accumulation and rectal distention.

**Figure 3:**
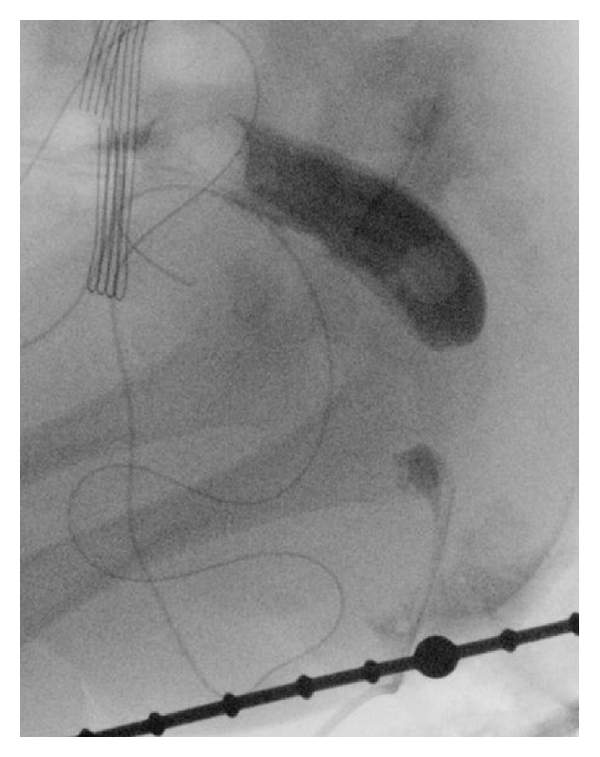
A cologram showed a distance of 2 cm between the rectal endings.

**Figure 4:**
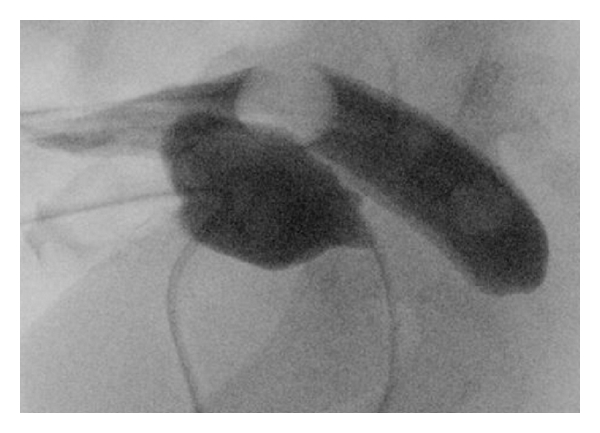
A combination of cologram and contrast in the urinary bladder excluded a fistula.

The child had no associated cardiac or urinary malformations. At 4 months of age, a cologram was carried out showing a blind ending of the rectum with no fistula. The distance between the endings was approximated to 2 cm (Figures 3 and 4). The urinary tract was examined with cystourethrogram before definitive surgery in order to disclose any urinary tract anomalies including urinary fistulas.

**Figure 5:**
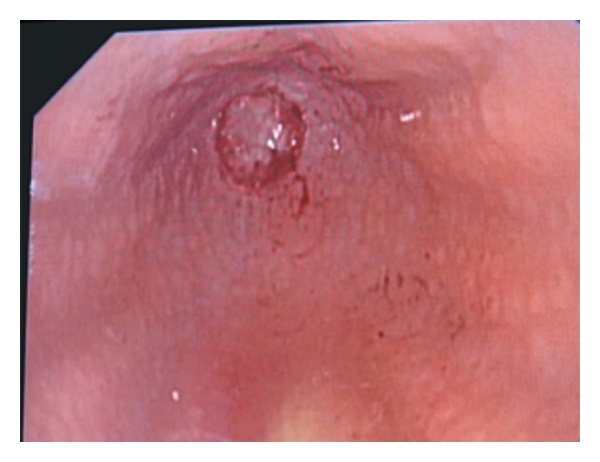
The endoscope was pushed into the passive stoma of sigmoidum. Here the bottom of the atresia is viewed. No fistula is seen.

**Figure 6:**
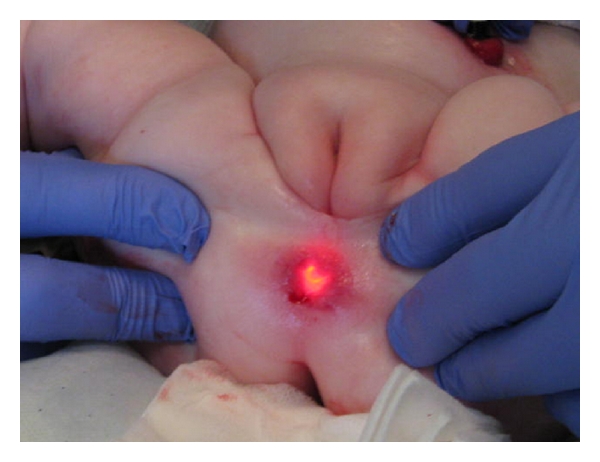
The endoscope was pressed against the rectal atresia, and with the help of external pressure, the endoscope could be seen 1 cm up in the anal channel.

**Figure 7:**
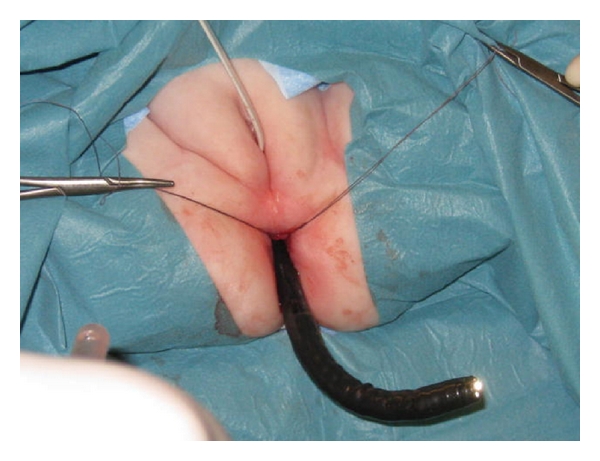
Holding stitches were set through the compressed 2 cm distance, and the blind ending was opened under excellent view. Then the endoscope could pass.

**Figure 8:**
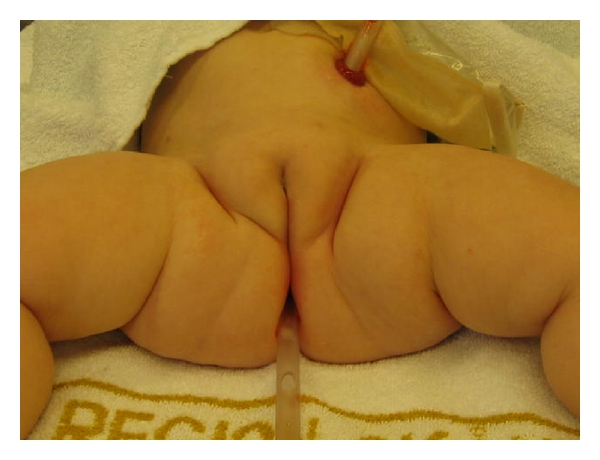
The anastomosis was completed with another 6 stitches of a monofile suture. Then a Foley catheter was placed for 7 days in order to avoid an immediate stricture.

The patient was operated on using the endoscopic and transanal approach. One of the operating surgeons passed a video endoscope (Olympus videoscope, 9 mm) through the sigmoidostomy down to the blind end of the rectum and telescoped through the distance of 2 cm and then almost protruded through the anus (Figures 5 and 6). The 2 cm thick wall between the endings of the rectum was divided with a diathermy between two stay sutures after which the coloscope came through the anus (Figure 7). The anastomosis was performed with a total of 6 stitches of resorbable suture material. In this case we chose to leave a Foley catheter 18 Ch which was left for 7 days through the anastomosis (Figure 8). This is not a routine. Two weeks after the operation, a daily dilatation schedule was initiated and continued until Hegar 16 could be used. No postoperative complications were seen.

**Figure 9:**
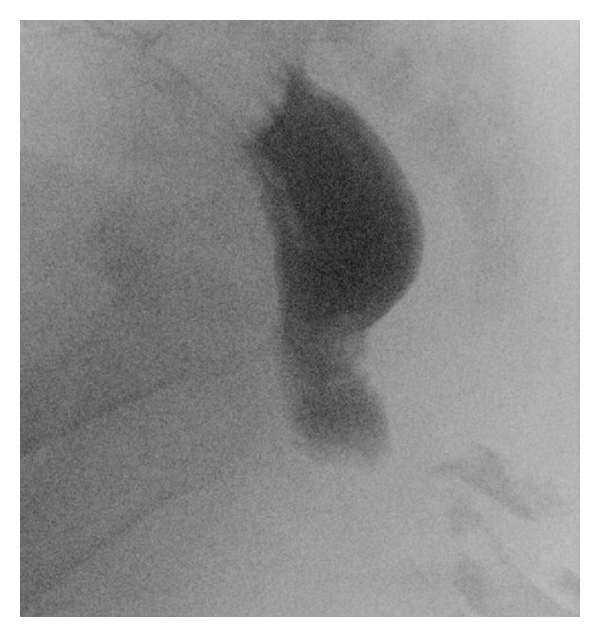
X-ray with contrast in the passive stoma. Before closure of the stoma, an open passage over the anastomosis was secured.

Closure of the sigmoidostomy was done when the patient was ten months old. Before closure, an X-ray of the rectum and anus was carried out to ascertain that there was no stricture (Figure 9). One and three months postoperatively the patient was in good condition and had one to three intestinal emptying daily. There had been no diarrhoea or sign of intestinal obstruction. The dilatations were continued once weekly with Hegar 16 in order to secure the good outcome.

## 3. Discussion

Rectal atresia is a rare condition, and there is no standardized recommended management in the literature. We have collected a list of the various reported operative procedures used for correction of this rectal malformation. These procedures are listed in Table 1 together with their references. The long list may reflect the inventive capacity of doctors worldwide and the difficulty faced in treating this anomaly because of its rarity and the variations in details. All the described methods seem to successfully help the patients to a normal life without incontinence or any serious sequel.

The operative management with the endoscopic and transanal approach protects and uses all elements contributed to faecal continence. The method is safe because of the good view, thanks to the light from the video-endoscope. The layers in between the rectal endings are pushed together under control, with both good visibility and working space so there is no risk of damage to nerves or other pelvic organs. In the child here reported the distance between the proximal and distal rectal ends was quite long, and therefore there should be a concern and care taken that the urethra could be pushed down and injured. A low rectal anastomosis can imply a certain risk of stricture. However, this can be avoided with regular rectal dilatation.

The use of endoscopy is associated with the presence of a colostomy. In neonates with rectal atresia and without colostomy PSARP is a good choice avoiding three surgical procedures necessary when using the approach here described. A similar procedure for imperforate anus without fistula has been described [4], using a needle knife to open the rectum from the inside at the exact point of convergence of the rectal columns. The perforation was made blindly through the subcutaneous tissue and muscular layers of the pelvic flour. This is a good idea regarding to where to place the anastomosis in children with anal atresia. This does not apply to children with rectal atresia and is not comparable with the procedure here described on children where the anus is intact and not a part of the anomaly and the rectal columns are not seen from above through the videoscope. Furthermore, the puncture was performed through an atresia tissue only. 

We found that the combination of endoscopy and transanal approach is a feasible alternative in the management of this rare condition. The operation is easy to do, and the risk of complications should be low since the anatomy is clearly visible. The followup of the child will include anal manometry as well as anal endoscopic ultrasonography [5].

##  Approvement

The publication of a paper on the child and X-ray finding and photography was approved by the child's guardians.

## Figures and Tables

**Table 1:** Several different techniques used for operating rectal atresia described in the literature.

The described methods	Reference number
Abdominoperineal pull-through	4
Sacroperineal pull-through	5
Duhamel's procedure	6
Stephen's posterior approach	6
Soave pull thorough	6
Posterior sagittal anorecto plastic (PSARP)	7, 8, 9
A string across the membrane using fluoroscopy	10
and progressively dilated the rectal canal	
Trans-anal end-to-end recto-rectal anastomosis	11
Magnetic compression anastomosis	12
Single stage sacral approach	13
TERPT (Transanal Endo-Rectal Pull-Through)	14
Laparoscopic + transanal approach	15
Abdominotransanal approach	16
Endoscopic and transanal here presented	
